# Natural Frequencies Identification by FEM Applied to a 2-DOF Planar Robot and Its Validation Using MUSIC Algorithm

**DOI:** 10.3390/s21041209

**Published:** 2021-02-09

**Authors:** Salvador Martínez-Cruz, Juan P. Amézquita-Sánchez, Gerardo I. Pérez-Soto, Jesús R. Rivera-Guillén, Luis A. Morales-Hernández, Karla A. Camarillo-Gómez

**Affiliations:** 1 Facultad de Ingeniería, Campus San Juan del Río, Universidad Autónoma de Querétaro, San Juan del Río, Querétaro 76807, Mexico; salvador.martinez.cruz@uaq.mx (S.M.-C.); jamezquita@uaq.mx (J.P.A.-S.); jesus.rooney.rivera@uaq.mx (J.R.R.-G.); luis.morales@uaq.mx (L.A.M.-H.); 2 Facultad de Ingeniería, Universidad Autónoma de Querétaro, Santiago de Querétaro, Querétaro 76010, Mexico; israel.perez@uaq.mx; 3 Tecnológico Nacional de México/Instituto Tecnológico de Celaya, Celaya, Guanajuato 38010, Mexico

**Keywords:** natural frequencies, finite element method, MUSIC algorithm, spectral analysis, 2-DOF planar robot

## Abstract

In this paper, the natural frequencies (NFs) identification by finite element method (FEM) is applied to a two degrees-of-freedom (2-DOF) planar robot, and its validation through a novel experimental methodology, the Multiple Signal Classification (MUSIC) algorithm, is presented. The experimental platforms are two different 2-DOF planar robots with different materials for the links and different types of actuators. The FEM is carried out using ANSYS™ software for the experiments, with vibration signal analysis by MUSIC algorithm. The advantages of the MUSIC algorithm against the commonly used fast Fourier transform (FFT) method are also presented for a synthetic signal contaminated by three different noise levels. The analytical and experimental results show that the proposed methodology identifies the NFs of a high-resolution robot even when they are very closed and when the signal is embedded in high-level noise. Furthermore, the results show that the proposed methodology can obtain a high-frequency resolution with a short sample data set. Identifying the NFs of robots is useful for avoiding such frequencies in the path planning and in the selection of controller gains that establish the bandwidth.

## 1. Introduction

During the last decade, robots, in particular those with servomotors, have gained momentum in diverse areas such as rehabilitation systems [1,2,3], human interaction systems [4], bio-inspired systems [5,6,7,8], and in surgery [9]. In general, their functionality depends on their performance trajectory; in this regard, an essential aspect to be considered in each programmed task is the value of the natural frequencies (NFs) of the robot, which can magnify the robot vibrations, affecting its performance [10,11,12,13,14]. Hence, it is of paramount importance to implement a method to accurately identify the NFs in order to obtain the best performance of the robot.

In literature, different strategies based on FEM and experimental procedures to estimate NFs in robots have been presented [15,16,17,18]. FEM is the most used experimental procedure for vibration measurements integrated with signal processing techniques because it can estimate the real behavior of robots [19,20,21,22,23,24]. Although FEM provides results that are very close-to-real [25], it is necessary to perform an experimental validation of the behavior of the robot to identify the NFs with accuracy [26]. An important step of the experimental procedure is the use of a signal processing technique to estimate the NFs of robots [22]. In this regard, Fourier-based methods [19,20,21,22,23] and wavelet transform (WT) [24,27] have been the most commonly employed methods for performing this task. For example, Min et al. employed the Fourier transform method to identify the NFs of an STR6-05 robotic arm, a 6-DOF heavy load manipulator, to develop a collision detection method based on vibration signals [22]. Similar work was presented by Yuan et al. [23], who applied Fourier transform to estimate the NFs of a robotic manipulator of 6 DOF in order to reduce its vibrations in a specific frequency range using a magnetorheological elastomer absorber on a spindle. Moreover, a swept sinusoidal signal for measuring the vibration response of the robot was used.

On the other hand, Chen et al. used the WT method for NFs identification of a simulated 4-DOF system subjected to forced excitations [28]. Klepka et al. applied the WT method for estimating the NFs of a simulated 2-DOF system [29]. The works presented above [19,20,21,22,23,24,27] have presented promising results for estimating the NFs of robots [19,20,21,22,23,24,27]; nevertheless, when a system or robot presents closed NFs, the methods presented are not efficient for identifying them [19]. Besides, Fourier-based methods are affected by noisy signals in complex systems and require a large number of samples [30], and an appropriate selection of the decomposition level and wavelet mother is necessary for WT to estimate the NFs appropriately [28]. For these reasons, it is essential to implement a signal processing technique for estimating the NFs of a robot under noisy signals with accuracy, especially closed NFs, using a small amount of data to avoid wear and tear on the robot during testing.

In recent years, a technique called the MUltiple Signal Classification (MUSIC) algorithm has provided promising results for analyzing induction motors [31,32], evaluating the behavior of civil structures [33,34,35], and impact-source-localization in composite structures under deformation conditions [36], among other applications. This technique presents diverse advantages such as noise immunity and high resolution and does not require a large amount of experimental information to estimate the frequencies contained in the analyzed signal with high accuracy [33]. It is also important to mention that the MUSIC algorithm provides an increased detectability of frequencies with a low amplitude as measured in robots [19,22,29], which is a significant advantage in this task. Hence, the MUSIC algorithm could provide an excellent alternative for estimating the NFs of robots.

This paper identifies the NFs of two different experimental platforms of a 2-DOF planar robot by FEM and its validation through the MUSIC algorithm. The FEM for two experimental platforms is presented. The experiment consists of an impulse-based trajectory applied to the end effector of the robot in order to excite it and obtain the response; then, the measured vibration signals are processed by the MUSIC algorithm and the fast Fourier transform (FFT) method [37] to identify the NFs. The experimental results show that the MUSIC algorithm can identify the NFs of the 2-DOF planar robot with a short sample data set and higher accuracy than the FFT method. Furthermore, the error between the MUSIC and the FEM results is lower than that obtained with FFT and the FEM results.

## 2. System Description

In this section, the general description of the two cases of study used in the experimentation and the dynamic model is presented.

### 2.1. Description of 2-DOF Planar Robot

**Figure 1:**
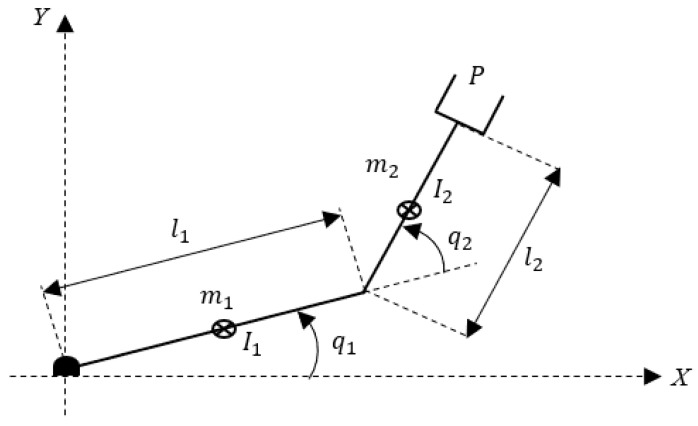
Simplified scheme of a 2-DOF planar robot.

Figure 1 shows the parameters of a 2-DOF planar robot with revolute joints that is evaluated in this work. The joint variable are represented by $q_{i}$, the length of the links is $l_{i},$ the link mass is $m_{i}$, the moments of inertia with respect to the *Z*-axis are denoted by $I_{i}$, for i=1,2, and the end effector is indicated by P.

**Figure 2:**
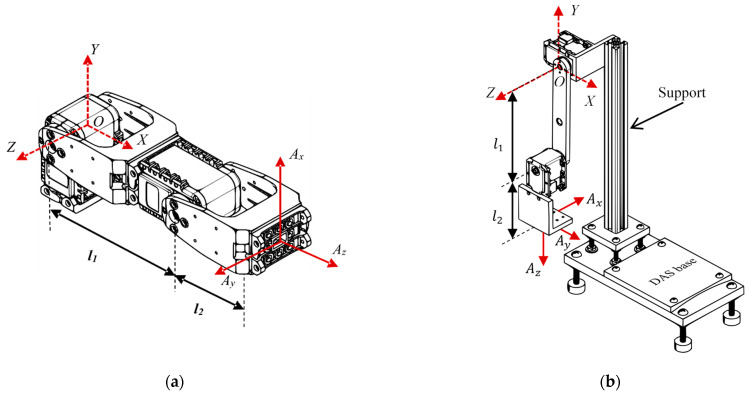
Experimental platforms of 2-DOF: (**a**) Case of study 1, and (**b**) Case of study 2 (The second case is designed and manufactured by the Engineering Faculty of the Universidad Autónoma de Querétaro, México).

The analyzed 2-DOF platforms in this work are presented in Figure 2, where the fixed reference frame has dashed lines and $A_{x},A_{y},A_{z}$ correspond to the accelerometer coordinate system attached to the end effector of the robot. Table 1 presents characteristics, such as the actuator models, material links, and weights of the two experimental platforms of 2-DOF. Table 2 presents the characteristics of the actuators, i.e., weight, dimension, and maximum torque, employed in the analyzed planar robots [38].

### 2.2. Dynamic Model

The simplified dynamic model of a 2-DOF robot is given by [39]:

(1) $$M\left(q\right)\ddot{q}+C\left(q,\dot{q}\right)\dot{q}+g\left(q\right)=\tau$$

where $q\in {\mathbb{R}}^{2}$ is the vector of the generalized joint coordinates, $\dot{q},\ddot{q}\in {\mathbb{R}}^{2}$ are the vectors of joint velocities and acceleration, respectively, $M\left(q\right)\in {\mathbb{R}}^{2\times 2}$ is the inertia matrix, $C\left(q,\dot{q}\right)\dot{q}\in {\mathbb{R}}^{2}$ is the vector of centrifugal and Coriolis forces, $g\left(q\right)\in {\mathbb{R}}^{2}$ is the vector of gravitational forces, and $\tau \in {\mathbb{R}}^{2}$ is a vector of forces applied by the actuators. The $C\left(q,\dot{q}\right)\dot{q}$, and g(q) vectors are obtained as follows:

(2) $$C\left(q,\dot{q}\right)\dot{q}=\dot{M}\left(q\right)\dot{q}-\frac{1}{2}\frac{\partial }{\partial q}\left[{\dot{q}}^{T}M\left(q\right)\dot{q}\right]$$

(3) $$g\left(q\right)=\frac{\partial u\left(q\right)}{\partial q}$$

where $\dot{M}\left(q\right)\in {\mathbb{R}}^{2\times 2}$ is the time derivative of the inertia matrix, while u(q)∈ℝ is the potential energy of the robot.

## 3. Natural Frequencies Identification

**Figure 3:**
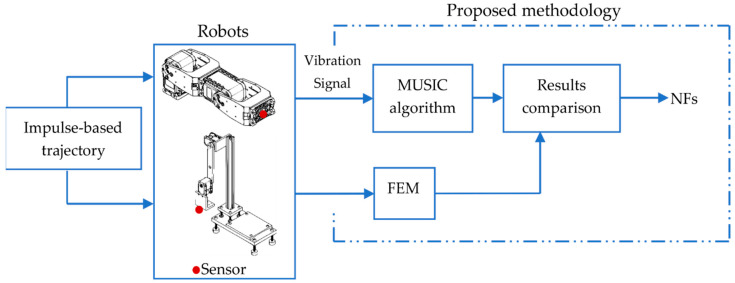
The schematic diagram for the proposed methodology.

Figure 3 shows the block diagram of the proposed methodology for estimating the NFs of a 2-DOF planar robot using the MUSIC method and FEM.

Firstly, an impulse-based trajectory was applied to the end effector of the robot in order to excite the mechanism. The trajectory consisted of 64 impulses applied to the end effector during an 8 s time window to each robot. The produced vibration signals were then acquired using a triaxial accelerometer and sent to a personal computer (PC) through a data acquisition system (DAS). Once the vibrations were measured, they were analyzed using the MUSIC algorithm to estimate the NFs of the 2-DOF robots. The FEM was performed for both 2-DOF robots in order to compare the obtained experimental results.

### 3.1. Multiple Signal Classification Algorithm

The MUSIC algorithm was first introduced by [40,41]; it belongs to the family of methods based on the decomposition of the observation space into signal and noise subspaces [25]. MUSIC algorithm considers that a signal x(t) is a sum of R complex sinusoids with a white noise additive, that is:

(4) $$x\left(t\right)=\overset{R}{\underset{k=1}{\sum }}A_{k}e^{j\left(2\pi f_{k}t+{ϛ}_{k}\right)+\omega \left(t\right)}$$

where $A_{k}$ is the amplitude of the sinusoid signal, $f_{k}$ is the frequency of the signal, ${ϛ}_{k}$ is the phase of the $k_{th}$ space vector, ω(t) is the white noise, and R is known as the MUSIC order. The sinusoid amplitude and frequency are not random or unknown. The phases of the sinusoids are uncorrelated random variables, uniformly distributed over the interval [−π,π]. The power spectrum of x(t) consists of a set of R impulses of area $2\pi \left|A_{k}\right|$ at frequencies $f_{k}$, for k=1,2,…,R, plus the power spectrum of the additive noise ω(t). Based on the orthogonality of the signal and noise subspaces, the MUSIC pseudospectrum $P_{MUSIC}$ of the current space, the vector, is given by the following frequency estimation function [42]:

(5) $$P_{MUSIC}\left(f\right)=\frac{1}{\sum_{i=P+1}^{M}{\left|{\bar{e}}_{i}{}^{H}{\bar{v}}_{i}\right|}^{2}}$$

where ${\bar{e}}_{i}{}^{H}$ is the signal vector defined as ${\bar{e}}_{i}{}^{H}\left(f_{i}\right)=\left[1,e^{-j2\pi f_{i}},\ldots ,e^{-j2\pi f_{i}\left(M-1\right)}\right]$, and ${\bar{v}}_{i}$ is the noise eigenvector. Equation (5) shows a maximum when, for a certain $f_{k}$ truly present in the signal, the signal and noise subspaces are zero.

### 3.2. MUSIC Verification Using Simulation Signals

**Figure 4:**
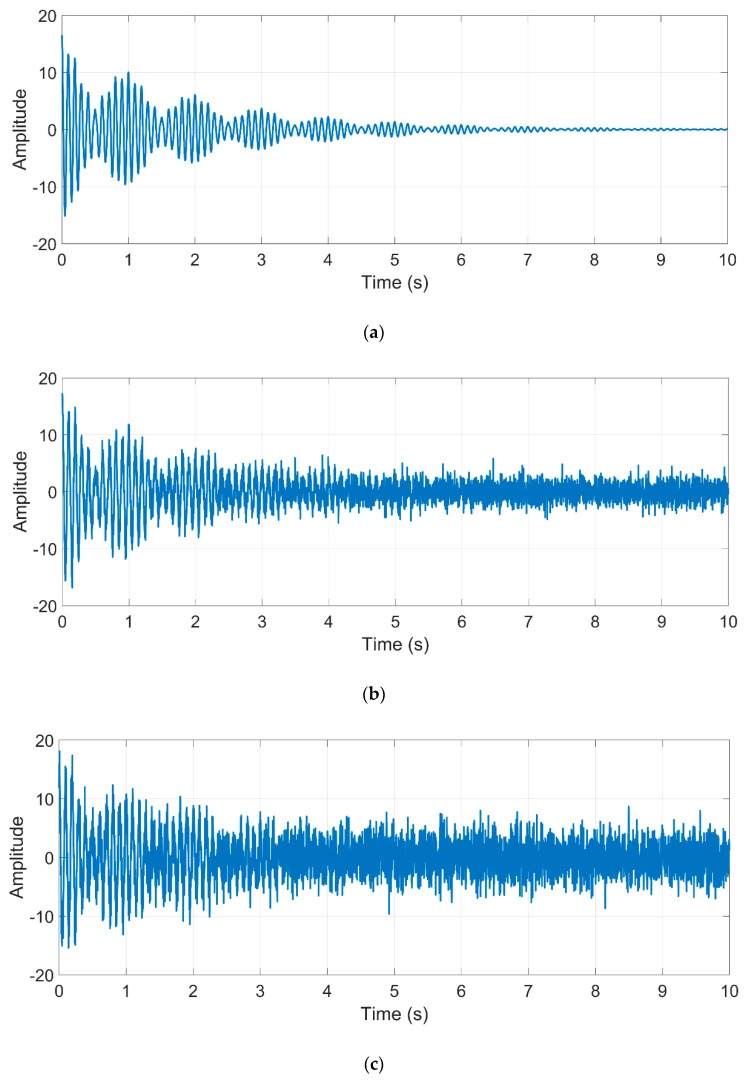
Simulated proposed signal (**a**) without noise, (**b**) with moderate-level noise, and (**c**) with high-level noise.

A numerical simulation was carried out to show the advantages of the MUSIC algorithm for identifying the frequencies of the time signal, in particular for the two closed ones, using few samples and a short sampling time. The FFT method was also employed to compare both methods to demonstrate the benefits of the proposed technique over the traditional FFT method. The analyzed signal simulates the free vibration of the robot with N modes. This is given by:

(6) $$j\left(t\right)=\overset{N}{\underset{i=1}{\sum }}e^{-t}A_{i}cos\left(2\pi f_{i}t+\varphi_{i}\right)$$

where $A_{i}$ is the respective amplitude of the *i*-th mode, $\varphi_{i}$ is the phase lag, and $f_{i}$ is the damped natural frequency. According to Equation (6), the synthetic signal is generated with the following parameters: N=4, meaning that there are four natural frequencies, $f_{1}=10$ Hz, $f_{2}=11$ Hz, $f_{3}=15$ Hz, $f_{4}=30$ Hz, and $A_{1}=10$, $A_{2}=5$, $A_{3}=1$, $A_{4}=0.9$, for the amplitudes. It uses a sampling frequency of $F_{s}=500$ Hz and a time window of T=10 s for generating a data set of 10,000 samples. Simulated proposed signals without noise, with moderate-level noise, and with high-level noise, are shown in Figure 4.

**Figure 5:**
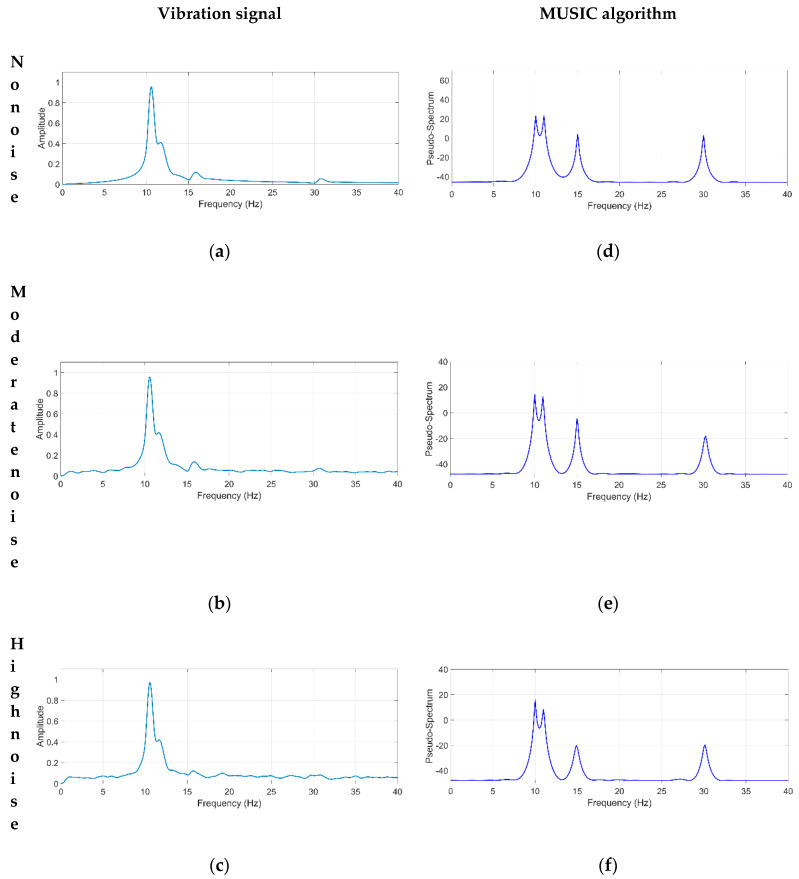
Spectra obtained by the FFT method for a signal with (**a**) no noise, (**b**) moderate-level noise, (**c**) high-level noise, and spectra obtained by the MUSIC method for a signal with (**d**) no noise, (**e**) moderate-level noise, and (**f**) high-level noise.

The influences of white-noise levels on identification accuracies were also considered. Figure 5 compares the NFs identification obtained by the MUSIC algorithm and that obtained by the traditional FFT method. During the analysis, two noise levels were considered in the signal: moderate-level (5dB) and high-level (0.01dB) noise, as well as a free of noise signal [43].

Furthermore, Figure 5a–c demonstrates that the FFT method is not capable of identifying the closed NFs, $f_{3}=10\;\mathrm{Hz}$ and $f_{4}=11\;\mathrm{Hz}$, due to their low-frequency resolution, which depends on the number of samples analyzed. Also, the frequency components corresponding to $f_{3}=15\;\mathrm{Hz}$ and $f_{4}=30\;\mathrm{Hz}$ are not clearly identified because of two main reasons: (1) the nonstationary nature of the signal (as measured in a robot), and (2) the noise contained in the signal [44].

On the other hand, the MUSIC algorithm with an order of 8 can identify the four NFs with high accuracy, as shown in Figure 5d–f. It is important to mention that the MUSIC method is not susceptible to noise and presents a high resolution, allowing accurate identification of frequency components in the analyzed signal, as demonstrated in Figure 5d–f. These advantages are reliable for identifying the NFs of robots since their responses are generally contaminated with high-level noise and present non-linear and nonstationary properties, as described by [22].

Table 3 summarizes the NFs estimated by the FFT and MUSIC methods and the error generated by both methods compared to the theoretical values. Observing Table 3, the maximum errors for NFs identification using the FFT method and the proposed MUSIC algorithm method are 6.6% and 1.66%, respectively. Hence, these results demonstrate clearly that the MUSIC algorithm is immune to noise and can identify the NFs accurately.

## 4. Numerical and Experimental Results

This section presents the numerical and experimental results for the two study cases. The numerical results were carried out by FEM using ANSYS™ software, and the experimental results were obtained by the MUSIC algorithm and the FFT method.

### 4.1. Finite Element Analysis

In the first case of study, the components meshed with four-node *solid tetrahedral elements* and the *hex-dominant method* contained in ANSYS™ software (shown in Figure 2a). The material properties used in this model were acrylonitrile butadiene styrene (ABS) polymer, with a mass density of ρ=1040 kg/m^3^, an elastic modulus E=2.045GPa, and a Poisson’s ratio v=0.35; and polybutylene terephthalate (PBT) polymer, with ρ=1340 kg/m^3^, E=1.93GPa, and v=0.3902. The model included 196,893 elements and 532,128 nodes.

The second case of study meshed mostly with four-node *solid tetrahedral elements*, and also with the *multizone method* applied in the support and link $l_{1}$ (shown in Figure 2b). The material used for this case was aluminum 6061 with the properties ρ=6280 kg/m^3^, E=69GPa, v=0.33; and ABS polymer material was used in the DAS base with the properties mentioned above. This model had a total of 310,733 elements and 559,653 nodes. In both cases of study, the links $l_{1}$ and $l_{2}$ were joined by the *bounded connection method*, and the reduction gears were not considered individually due to computational complexity. However, in the finite element model, the actuators were considered as a single body whose mass and volume coincided with the real actuator.

**Figure 6:**
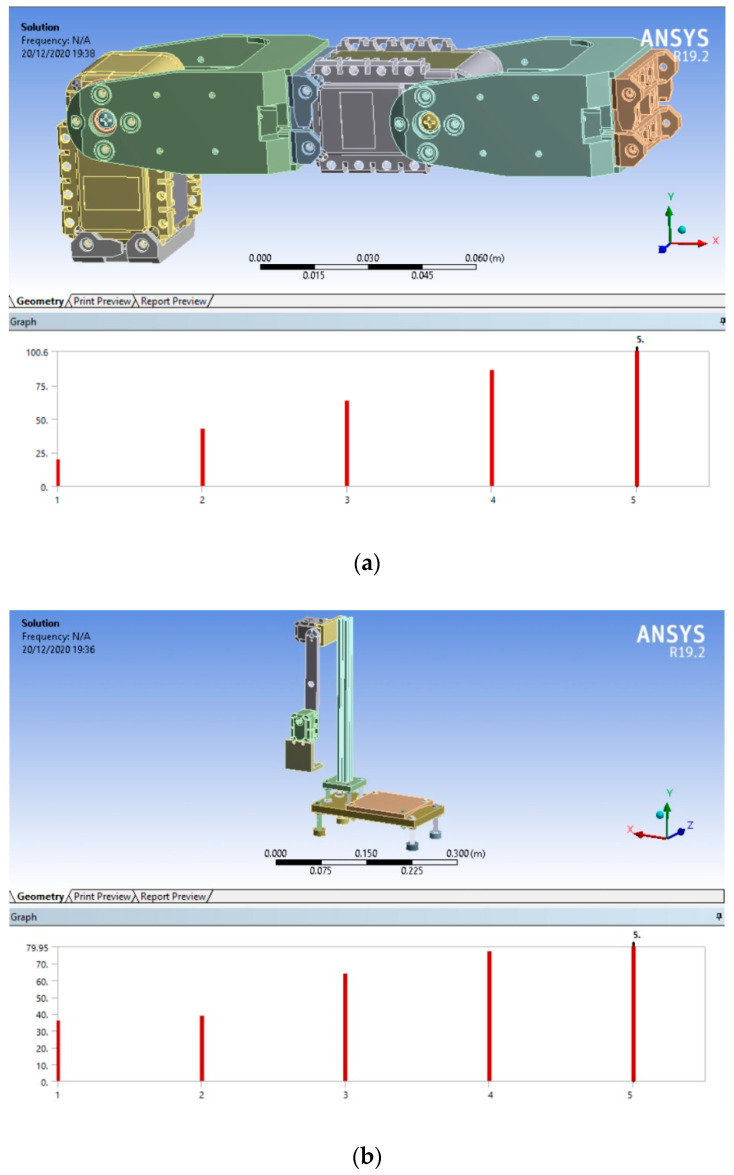
ANSYS™ results: (**a**) case of study 1; (**b**) case of study 2.

After setting the parameters and constraints of the numerical model, a FEM is applied to each case of study with the position shown in Figure 2 to calculate the NFs for each robot. The numerical results obtained by ANSYS™ are presented in Figure 6. It should be mentioned that the path of the end effector of the robot does not reach frequencies higher than 110 Hz [20,23,24]; hence, in this paper, the first five NFs of the robot are considered, which are inside this frequency range.

Figure 6a shows the first five NFs of the 2-DOF planar robot, which corresponds to the first case of the study. The obtained NFs values are 19.765 Hz, 42.622 Hz, 63.427 Hz, 86.386 Hz, and 100.580 Hz, which correspond with the frequencies that could be excited with the commonly used trajectories in this type of robot and result in undesirable robot performance and lack of stability due to the resonance effect. Figure 6b presents the first five NFs for the second case of study, where closed frequencies can appear because of the geometrical effects of the aluminum structure, such as symmetry and similar physical properties [45,46]. The obtained NFs values are 35.987Hz, 38.866Hz, 63.736Hz, 77.029Hz, and 79.952 Hz. These results are subsequently validated with the proposed methodology based on the MUSIC algorithm to identify NFs in the two cases of study.

A summary from the FEM analysis results of Figure 6 for the two cases of study is shown in Table 4. As observed in Table 4, the NFs corresponding to the case of study 1 are separated from each other. However, the case of study 2 presents closed frequencies, which can be associated with the geometrical effects of the aluminum structure [45,46]. Hence, the following step in the proposed methodology is to experimentally estimate the NFs with FFT and MUSIC methods.

### 4.2. Experimental Setup

**Figure 7:**
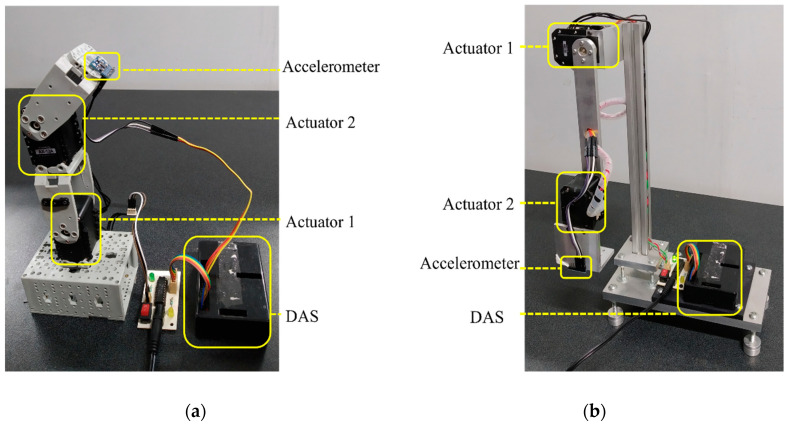
Experimental Setup: (**a**) 2-DOF planar robot with two Dynamixel AX-12 servomotors; (**b**) 2-DOF planar robot with two Dynamixel MX-28 servomotors.

In the experiment, the vibration signal was acquired to identify the NFs of the robots. In both cases, an InvenSense model MPU-6050 triaxial accelerometer was used for measuring the vibration signals of robots, which was placed on the end effector of the robot (shown in Figure 7). The accelerometer had a user-selectable full scale of ±2g,±4g,±8g, and ±16g (g=9.81 m/s^2^), with a resolution of $5\times 10^{-4}g$ over a 100Hz bandwidth. A DAS composed of an Atmel microcontroller and a 16-bit analog-to-digital converter was employed to acquire the vibration signals. The vibration signals measured by the accelerometer were stored in the DAS and sent to a PC using the USB protocol. The DAS used a sampling frequency of 1kHz during a time window of 8 s for every test, obtaining 8000 samples for each test.

Figure 7 shows the overall experimental setup for estimating the NFs of both robots. In order to obtain the vibration response of the robots, they were excited using an impulse-based trajectory. The trajectory was applied to the end effector of the robots, and consisted of 64 impulses over 8 s. Figure 7a shows the first experimental setup, which consists of a 2-DOF planar robot comprised of two servomotors from Dynamixel model AX-12 as actuators. These servomotors worked with a half-duplex communication protocol, a baud-rate of 1Mbps, a resolution of 10bits(0−1023), and a maximum speed of 114 RPM. The second experimental setup is shown in Figure 7b, where a 2-DOF planar robot with a different configuration is analyzed, and includes two Dynamixel model MX-28 servomotors. They also work with a half-duplex communication protocol, a baud-rate of 1 Mbps, a resolution of 12bits(0−4095), and a maximum speed of 116.62 RPM. For the experiments, the two robots (presented in Figure 7) were fixed to a worktable by screws to avoid undesirable vibrations that were not typical of the robot.

### 4.3. Experimental Results

This section presents the experimental results, which are compared with the FFT method to show the effectiveness of the proposed method.

**Figure 8:**
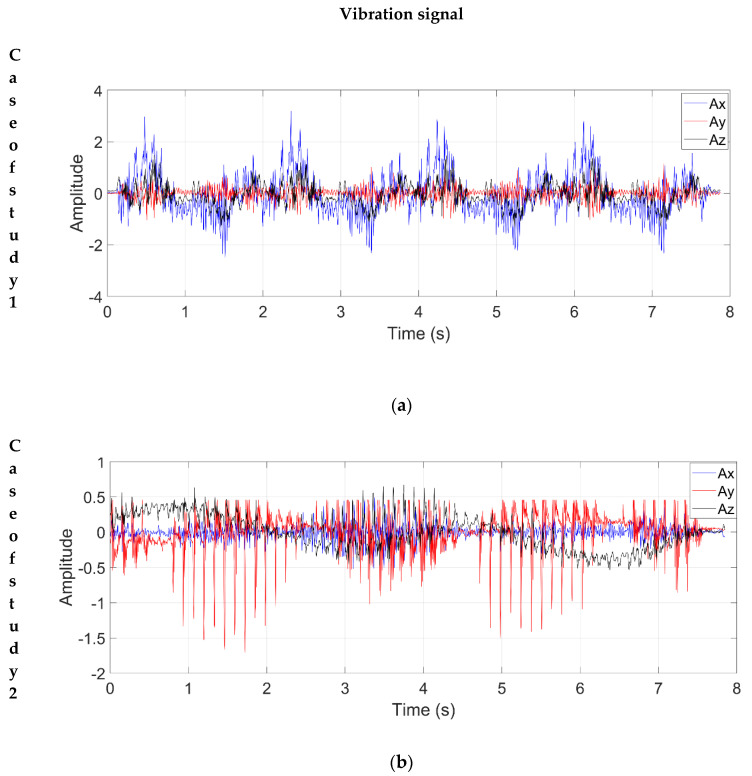
Vibration signal: (**a**) Case of study 1; (**b**) Case of study 2.

According to the proposed methodology, the vibration responses of both robots were measured by applying an impulse-based trajectory, which was repeated ten times in both cases. The signals were acquired using the same conditions, i.e., the same trajectory of the end effector, the same 64 impulses, and a time window of 8 s. Figure 8a,b shows an example of the vibration responses measured by the triaxial accelerometer, Ax,Ay, and Az, for each 2-DOF planar robot, respectively.

**Figure 9:**
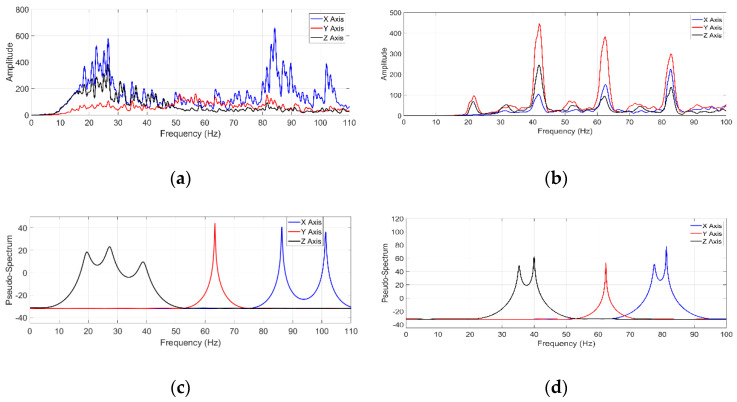
Signal processing results: (**a**) FFT spectrum for case of study 1; (**b**) FFT spectrum for case of study 2; (**c**) MUSIC spectrum for case of study 1; (**d**) MUSIC spectrum for case of study 2.

Once the vibration signals were acquired, the processing was carried out using the MUSIC algorithm and the FFT method to make a comparison. Figure 9 shows the spectra for the two cases of study; Figure 9a,b corresponds to the FFT method, while Figure 9c,d corresponds to the MUSIC algorithm.

According to the obtained spectra, the FFT method cannot identify the closed NFs of a robot because it is sensitive to noise. As shown in Figure 9a,b, the FFT method presents a large number of undesirable peaks due to the amount of noise present in the signal and fails to accurately discern the frequency components corresponding to the NFs of the robot. Hence, the FFT method is susceptible to diverse problems when it is used for analyzing noisy signals with nonstationary properties as measured in the robots, such as the appearance of spurious frequencies, as observed in Figure 9a,b, because of the spectral leakage, resolution, and the high-level of noise contained in the analyzed signals (see Figure 8a,b) [47].

On the other hand, the MUSIC algorithm can identify the NFs of a robot even when the signal contains a high-level noise. Figure 9c shows that the MUSIC algorithm can accurately estimate the NFs since it is not affected by noise, which enables more accurate identification of the NFs compared with the FFT method. Additionally, Figure 9d shows that the proposed method can identify the closed frequencies in the second case of study with high accuracy. Unlike the FFT method, the proposed method can identify low-amplitude frequencies, which is advantageous because the NFs of a robot can have a small amplitude.

As shown in Figure 9c,d, the MUSIC algorithm can identify the NFs with higher frequency-resolution than the FFT method, even when the signal is embedded in a high-level noise and with a short sample data set. Table 5 shows the values of the first five identified NFs by the proposed methodology in the two cases of study.

## 5. Results and Discussion

Table 6 shows the values of the first five identified NFs by the FFT method and the MUSIC algorithm, as well as their corresponding analytical values obtained by FEM for the two cases of study. As it is shown, the MUSIC algorithm can identify the NFs at a very similar level when compared with the analytical calculation from FEM. Besides, the MUSIC algorithm does not require a long time window to provide a high-frequency resolution, avoiding wear on the actuators during testing through a short robot testing time. On the other hand, the FFT method presents a significant difference compared to FEM due to the noise present in the signal; this is a significant advantage of the proposed methodology because most of the real signals have a considerable noise level.

**Figure 10:**
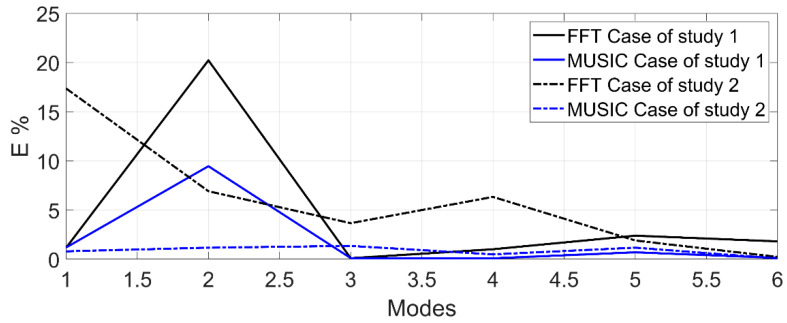
Error percentage of the results from FFT and MUSIC compared to the analytical results from FEM.

Figure 10 shows the percentages of similarity between the results of the MUSIC algorithm and FFT method against FEM. These values are obtained by:

(7) $$E\%=\frac{abs\left(f-r\right)}{r}\times 100$$

where f is the analytical calculation results, r is the experimental results (MUSIC or FFT), and E% is the error percentage for the corresponding modes.

Observing Figure 10, the results of the FEM analysis by ANSYS™ using the two cases of study provide an acceptable accuracy compared with experimental results obtained with the MUSIC algorithm, i.e., the results of FEM are close-to-real, with a maximum error of 9.46% for the case of study 1 (denoted by the solid blue line) and 3.44% error for the case of study 2 (denoted by the dotted blue line). However, if the FEM results are validated using the experimental results of the FFT method, the percentage error is more significant, at 20.22% (denoted by the solid black line) and 11.14% (denoted by the dotted black line) for the cases of study 1 and 2, respectively. In this regard, the proposed methodology can contribute to the planning of the trajectories of the robot. It will also be useful for the selection of controller gains to avoid exciting the robot in the NFs and assist in the correct selection of notch filters at the output of a controller.

Notice that the MUSIC algorithm requires the previous selection of the algorithm order, which is chosen according to the number of frequencies or components found in the analyzed signal. In this regard, the number of frequencies contained in the signal is unknown, but it is known that the trajectories used in this type of robot are in the range from 0 Hz to 110 Hz [20,23]. In this sense, the FEM results allow the selection of the MUSIC algorithm order because they provide the number of natural frequencies contained in the range of interest (0Hz to 110Hz), showing that an order of 10 is the most reliable for identifying the main NFs contained in the signal. On the other hand, the MUSIC algorithm presents more computational complexity than the FFT; however, it is superior to the FFT method for identifying the NFs of both robots under noisy signals. The numbers of operations for computing the FFT (OpFFT) [48] and MUSIC (OpMUSIC) [49] are given by the following equations:

(8) $$OpFFT=N{log}_{2}N$$

(9) $$OpMUSIC=N^{3}$$

where N represents the data length.

## 6. Conclusions

This paper presents a novel methodology to identify NFs in 2-DOF planar robots, a FEM is applied in two cases of study, and the results are validated with a vibration signal analysis through the MUSIC algorithm. Two cases of study are modeled in ANSYS™ software, and a FEM is applied to estimate the NFs of the robots. Also, a simulated signal is analyzed to show the effectiveness of the MUSIC algorithm. The experimentation consists of an impulse-based trajectory applied to the end effector of the robot to excite the mechanism; the vibration signals are measured by a triaxial accelerometer and processed by the proposed methodology and the FFT method to show the advantages of the MUSIC algorithm compared with the traditional method. The experimental results show that the MUSIC algorithm is closer to the FEM results, and it is a useful methodology for NF identification in 2-DOF planar robots because it is not affected when the signal is contaminated with a high-level noise. In this context, the MUSIC algorithm is advantageous and has a higher resolution than traditional Fourier-based methods. The experimental cases are carried out in an 8 s time window. Hence, the simulated and experimental results show that the MUSIC algorithm does not need a long sample time window to obtain a high-frequency resolution. The proposed methodology has been developed in a 2-DOF planar robot without loss of generality; that is, the instrumentation and signal acquisition process is the same for an *n*-DOF robot arm. The MUSIC algorithm can also contribute to path planning of the robot, the selection of gains of a controller to avoid exciting the robot in the NFs, and the correct selection of notch filters at the output of a controller.

## Figures and Tables

**Table 1:** Characteristics of the two experimental platforms of 2-DOF.

	Case of Study 1	Case of Study 2
Actuators	Dynamixel AX-12	Dynamixel MX-28
Material link	Polymeric	Aluminum 6061
Total weight	0.16 kg	0.7475 kg
$l_{1}$	0.093 m	0.14 m
$l_{2}$	0.081 m	0.081 m
$m_{1}$	0.0219 kg	0.0566 kg
$m_{2}$	0.0219 kg	0.0712 kg
$I_{1}$	$8.317\times 10^{-6}$kg·m^2^	$1.11\times 10^{-4}$kg·m^2^
$I_{2}$	$8.317\times 10^{-6}$kg·m^2^	$3.59\times 10^{-5}$kg·m^2^

**Table 2:** Properties of the actuators.

	Dynamixel AX-12	Dynamixel MX-28
Weight	0.0546 kg	0.072 kg
Dimensions	0.032 m × 0.05 m × 0.04 m	0.0356 m × 0.0506 m × 0.0355 m
Max. torque	1.52 N·m	2.5 N·m

**Table 3:** Natural frequencies (NFs) identified by FFT and MUSIC methods.

Mode	Theoretical Frequency (Hz)	FFT Method (Error %)			MUSIC Method (Error %)		
		WN	MLN	HLN	WN	MLN	HLN
1	10	10.3 (3)	10.5 (5.5)	10.5 (5.5)	10 (0.0)	10.01 (0.1)	10.01 (0.1)
2	11	NI	NI	NI	11 (0.0)	10.98 (0.18)	10.98 (0.18)
3	15	16 (6.6)	15.8 (5.3)	NI	15 (0.0)	15.01 (0.06)	15.25 (1.66)
4	30	31 (3.3)	NI	NI	30 (0.0)	30.27 (0.9)	32.34 (1.06)

NI: not identified; WN: without noise; MLN: moderate-level noise; HLN: high-level noise.

**Table 4:** Summary of simulation results.

	Natural Frequencies (Hz)	
Mode	Case of Study 1	Case of Study 2
1	19.765	35.987
2	42.622	38.866
3	63.427	63.736
4	86.386	77.029
5	100.580	79.952

**Table 5:** Experimental results for the MUSIC algorithm and the FFT method.

	Natural Frequencies (Hz)			
Mode	Case of Study 1		Case of Study 2	
	MUSIC	FFT	MUSIC	FFT
1	19.53	20	34.79	40.5
2	38.94	34	38.33	40.5
3	63.35	63.5	62.99	61.5
4	86.3	85.5	77.03	81.5
5	101.3	103	80.93	81.5

**Table 6:** Comparison of natural frequencies.

Natural Frequencies (Hz)						
	Case of Study 1			Case of Study 2		
Modes	FEM	FFT	MUSIC	FEM	FFT	MUSIC
1	19.765	20	19.53	35.987	40.5	34.79
2	42.622	34	38.94	38.866	40.5	38.33
3	63.427	63.5	63.35	63.736	61.5	62.99
4	86.386	85.5	86.3	77.029	81.5	77.03
5	100.580	103	101.3	79.952	81.5	80.93

## Data Availability

Not applicable.
